# Effects of low birth weight on time to BCG vaccination in an urban poor settlement in Nairobi, Kenya: an observational cohort study

**DOI:** 10.1186/s12887-015-0360-5

**Published:** 2015-04-18

**Authors:** Martin Kavao Mutua, Rhoune Ochako, Remare Ettarh, Henrik Ravn, Elizabeth Echoka, Peter Mwaniki

**Affiliations:** African Population and Health Research Center, Manga Close, Nairobi, Kenya; Research Center for Vitamins and Vaccines, 5 Artillerivej, Copenhagen, Denmark; Center for Public Health Research, Kenya Medical Research Institute, Nairobi, Kenya; Jomo Kenyatta University of Agriculture and Technology, Nairobi, Kenya; Faculty of Medicine, University of British Columbia, Vancouver, Canada; Population Services Kenya, Jumuia Place, Lenana road, Nairobi, Kenya; Bandim Health Project, Statens Serum Institut, 5 Artillerivej, Copenhagen, Denmark; OPEN, University of Southern Denmark/Odense University Hospital, Odense, Denmark

**Keywords:** Bacillus Calmette-Guérin, Low birth weight, Immunization

## Abstract

**Background:**

The World Health Organization recommends Bacillus Calmette-Guérin (BCG) vaccination against tuberculosis be given at birth. However, in many developing countries, pre-term and low birth weight infants get vaccinated only after they gain the desired weight. In Kenya, the ministry of health recommends pre-term and low birth weight infants to be immunized at the time of discharge from hospital irrespective of their weight. This paper seeks to understand the effects of birth weight on timing of BCG vaccine.

**Methods:**

The study was conducted in two Nairobi urban informal settlements, Korogocho and Viwandani which hosts the Nairobi Urban Health and Demographic Surveillance system. All infants born in the study area since September 2006 were included in the study. Data on immunization history and birth weight of the infant were recorded from child’s clinic card. Follow up visits were done every four months to update immunization status of the child. A total of 3,602 infants were included in this analysis. Log normal accelerated failure time parametric model was used to assess the association between low birth weight infants and time to BCG immunization.

**Results:**

In total, 229 (6.4%) infants were low birth weight. About 16.6% of the low birth weight infants weighed less than 2000 grams and 83.4% weighed between 2000 and 2490 grams. Results showed that, 60% of the low birth weight infants received BCG vaccine after more than five weeks of life. Private health facilities were less likely to administer a BCG vaccine on time compared to public health facilities. The effects of low birth weight on females was 0.60 and 0.97-times that of males for infants weighing 2000–2499 grams and for infants weighing <2000 grams respectively. The effect of low birth weight among infants born in public health facilities was 1.52 and 3.94-times that of infants delivered in private health facilities for infants weighing 2000–2499 grams and those weighing < 2000 grams respectively.

**Conclusion:**

Low birth weight infants received BCG immunization late compared to normal birth weight infants. Low birth weight infants delivered in public health facilities were more likely to be immunized much later compared to private health facilities.

## Background

The World Health Organization (WHO) recommends Bacillus Calmette-Guérin (BCG) immunization against tuberculosis (TB) at birth or at first clinical contact to all infants [1] with the exception of HIV-infected infants in whom the BCG vaccine is associated with significant safety concerns [2]. Approximately nine million new cases of TB were diagnosed in 2013, with 1.5 million deaths reported. Most of these cases and deaths occurred in low and middle income countries [3], Kenya is among the 22 high burden TB countries with an estimated incident rate of 268 cases per 100,000 [3]. In Kenya, the Ministry of Health through the Division of Vaccines and Immunization recommends that the BCG vaccine be given at birth or at first clinical contact, except for pre-term and low birth weight (LBW) infants (birth weight less than 2000 grams) who should be vaccinated at the time of discharge from hospital irrespective of the weight [4]. Overall, BCG coverage in Kenya is high (96%) [5], but little is known about how soon the vaccines should be given after birth. BCG immunization delays have been reported in other studies [6,7] even in settings where the coverage is high [8].

When immunization is delayed, there is an increased risk or severity of infections during infancy due to the shortening of the duration of the protective effect of the vaccines [8]. Studies in West Africa have shown BCG immunization to have beneficial non-specific effects (NSE)–effects other than protecting the child from tuberculosis infections [9,10]. The earlier BCG immunization is given to an infant the higher the chances of survival [11-14]. While many studies have looked at timeliness of selected vaccines, few have focused on timeliness of vaccine administration in African settings. Moreover limited literature on timing of BCG among LBW infants is available. This paper, seeks to document the effects of LBW on timeliness of BCG immunization in two urban informal settlements in Nairobi, Kenya where healthcare access through the public sector is limited.

## Methods

### Study setting

The study was carried out in two informal settlements of Nairobi (Viwandani and Korogocho) where the African Population and Health Research Center (APHRC) runs the Nairobi Urban Health and Demographic Surveillance System (NUHDSS). The NUHDSS has been in operation since 2002 and had about 81,129 registered inhabitants in nearly 31,977 households as of December 2012. These two densely populated communities have high unemployment, poverty, crime, poor sanitation and generally poorer health indicators when compared to Nairobi as a whole [15,16]. The two communities however have notable differences: Viwandani is bordered by an industrial area and attracts migrant workers with relatively higher education levels, while the population in Korogocho is more stable and shows more co-residence of spouses. In addition, Korogocho has less disparity with regard to gender and age distribution of the population compared to Viwandani.

### Study population

**Figure 1:**
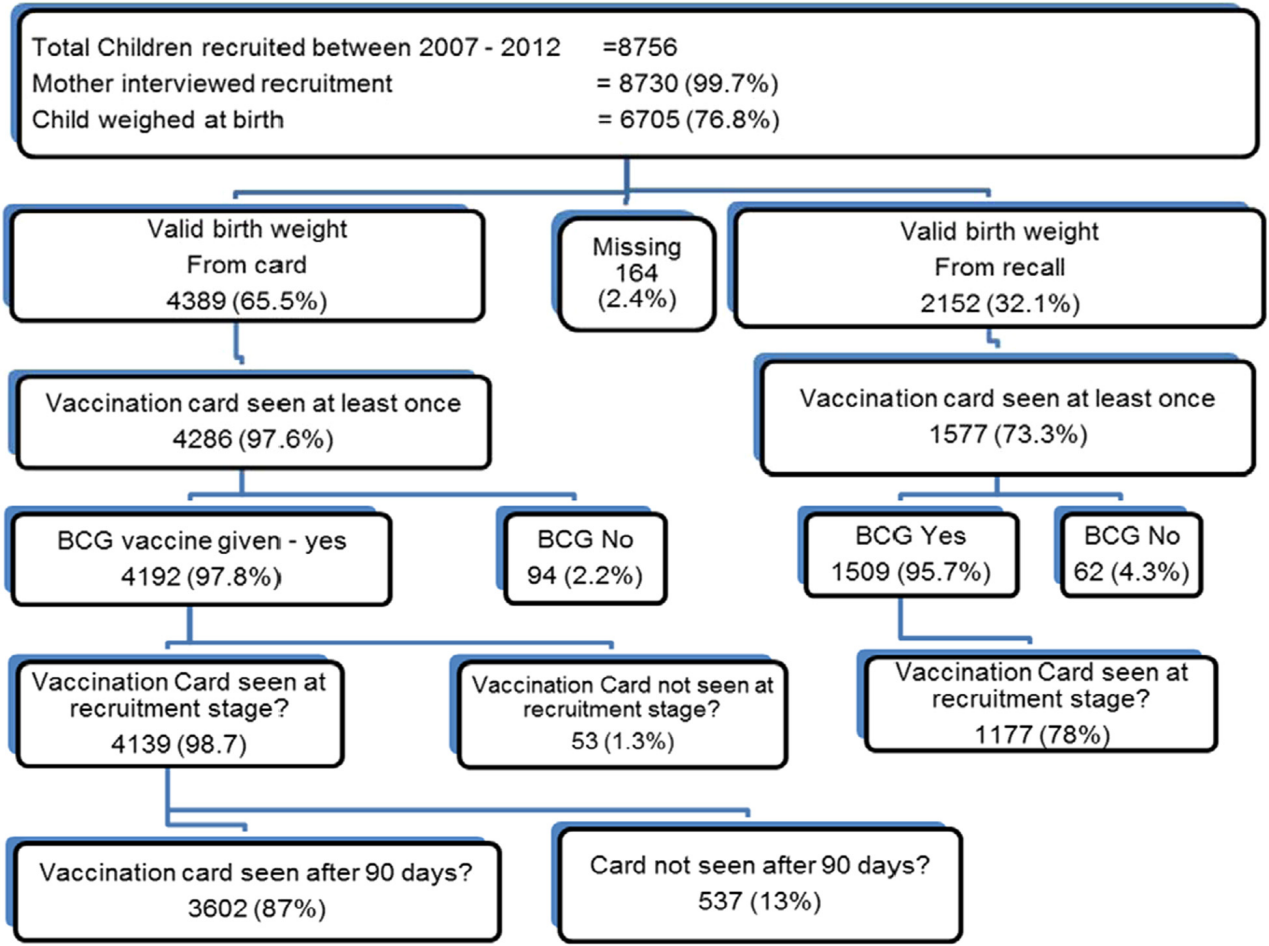
Derivation of the sample of infants included in the study from the NUHDSS.

This study used maternal and child health data collected at the NUHDSS from two periods of survey: The first period 2007–2010 under “Urbanization, Poverty and Health Dynamics” (UPHD) project funded by Wellcome Trust that recruited all infants born from September 2006 and the second period 2011–2013 funded by Danish Development Agency (DANIDA) through INDEPTH Network and recruited infants born from January 2010. In addition to following up infants from the first period. During both periods, mother-child pairs were recruited and followed up with visits every four months as applicable. The same questionnaire was administered during each visit by trained interviewers. Information on place of delivery, child’s weight at birth was collected during the first visit, while the children’s immunization history including BCG vaccine given at birth collected during the first three visits. A total of 8,756 infants were recruited with 34,969 visits in total during the period of the study. Not all infants were seen during follow up visits due variety of reasons which included deaths, out migration, temporary exits among others. A breakdown of the selected sample for analysis is given in Figure 1. For this study, 3,602 infants (1668 and 1934 from first and second period) for whom an immunization card with a valid BCG vaccine date was seen at least once after 90 days of age and a birth weight recorded during the first visit were included. Only infants who had a BCG immunization and survived more than 90 days were included in the study because they had at least two additional opportunities where the child could have received BCG immunization at 6 and 10 weeks old during the child’s visit for the first and second doses of Polio and Pentavalent immunizations.

### Variables

Our outcome variable of interest is age at BCG immunization. The weight at birth of the child (recorded from a health card) was used as the primary exposure variable. We defined low birth weight (LBW) and normal birth weight (NBW) as a infants weighing less than 2500 grams and more or equal to 2500 grams respectively [17,18]. For purposes of this analysis, we sub-classified low birth weight to those weighing below 2000 grams and those weighing 2000–2499 grams. Other variables such as; Mother educational level (incomplete/no education, completed primary education or secondary school or above), Place of Delivery (Public or Private), Ethnicity (Kikuyu, Luhya, Luo, Kamba or Others), Settlement area (Korogocho or Viwandani), Child’s Gender (Male or Female) and Pregnancy Intention of the index child (Wanted the child at that time, Wanted the child later or did not want the child at all) were used as control variables.

### Data analysis

**Figure 2:**
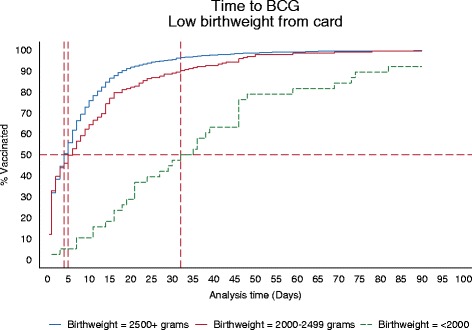
Kaplan-Meier, Cox and lognormal curves for the different categories of the child’s birth weight.

Descriptive statistics, frequencies, proportions and median of all dependent and independent variables of interest were obtained to summarize the data. The main objective was to assess the age when a child is vaccinated with BCG, and compare LBW infants with the normal birth weight infants. Approximately 1% of the infants in the study received their BCG after 90 days; these were censored at 90 days of age. Thus, Survival analysis techniques were used to assess the time to BCG immunization before 90 days. Kaplan-Meier curves were used to describe the age distribution of getting BCG for the three birth weights group. Cox regression was later used to estimate the impact of low birth weight on the timing of the BCG immunization and identify risk factors of delay to BCG immunization; Cox models assumes proportionality of the hazard function over time. The proportionality assumption did not hold for our data, this was deduced using the Kaplan-Meier curves (Figure 2) and tested statistically using Schoenfeld residuals. Therefore we fitted parametric regression model methods that allow for the baseline hazards to vary for different categories for an independent variable and the hazard function does not need to be proportional. Different distributions for the baseline hazards were considered (exponential, Weibull, Gompertz, Lognormal, Log logistics and gamma). Akaikes information criterion (AIC) was used to pick the best distribution (model) which fits our data well. The AIC suggested a log normal accelerated failure time (AFT) model. Log normal parametric model was thus used to assess the association between LBW and time to BCG immunization.

Unadjusted (*Model 1*) and adjusted (*Model 2*) time ratios (TR) were used to assess the association between birth weight and BCG timing. Time ratio compares the change in the survival time associated with a change in the values of a given covariate. In an AFT model, every subject has the same “baseline” survival curve, the covariates effects serve to accelerate the passage of time.

Interaction models (*Model 3 and 4*) were fitted to see how the birth weight interacts with child’s gender and place of delivery (Public or Private Health facility) respectively.

### Ethical considerations

The study was approved by Kenya Medical Research Institute (KEMRI) ethical review committee with annual renewals during the second phase of the study. The research assistants were trained on research ethics and obtained written and verbal informed consent from all the study respondents. The NUHDSS, on which the study was nested, also received ethical approval from KEMRI’s Ethical Review Board.

## Results

### Descriptive statistics

A total of 229 out of 3602 infants (6.4%) included in the analysis were LBW (see Table 1). Of these, 38 (16.6%) infants weighed below 2000 grams with a median age at BCG immunization of 34 days interquartile range [IQR] 18–46, 191 (83.4%) weighed between 2000–2499 grams with a median immunization age of 6 days IQR 1–15. The remaining 93.6% had normal birth weight with median age of 4 days IQR 1–10. Infants from Korogocho (52% of the sample) on average were receiving the BCG a day later (median 5 days IQR 1–11) than those from Viwandani (median 4 days IQR 1–10). In terms of mother’s education level, the less educated (incomplete or no education) received the BCG vaccine a day later as compared to those who completed primary or higher (median 5 days IQR 1–12 compared to 4 days IQR 1–10) education. In general, infants delivered in a health facility received their BCG earlier (median 4 days IQR 1–10), as expected, compared to infants delivered at home or by a traditional birth attendant (median days IQR 5–16). Additionally, infants delivered at a public health facility had a median age of 1 day IQR 1–7 compared to those delivered at a private health facility (median 6 days IQR 2–11). Males received BCG immunization a day later (median 5 days IQR 1–11) as compared to females (median 4 days IQR 1–10). About 72 infants (2%) of all infants in the study received BCG vaccine together with the first dose of the pentavalent vaccine. Infants who received BCG with the first dose of pentavalent were more likely to weigh less than 2000 grams (21%) compared to normal birth weight infants (2%) and infants weighing 2000–2499 grams (3%). Among infants excluded from the analysis due to lack of BCG information from their immunization cards, 93% had normal birth weight, 4% weighed 2000–2499 grams and 3% weighed below 2000 grams.

**Table 1:** **Sample summary statistics**

	**N**	**%**	**Median age (IQR) of BCG vaccination**
Child’s birth weight (grams)			
<2000	38	1.1	34 (18–46)
2000_2499	191	5.3	6 (1–15)
> = 2500	3,373	93.6	4 (1–10)
Settlement area			
Korogocho	1,881	52.2	5 (1–11)
Viwandani	1,721	47.8	4 (1–10)
Mothers level of education			
Incomplete primary/no education	910	26.4	5 (1–12)
Completed primary	1,564	45.2	4 (1–10)
Secondary+	983	28.4	4 (1–10)
Place of delivery			
Non health facility	136	3.8	9 (5–16)
Health facility	3,466	96.2	4 (1–10)
Type of Health Facility			
Private	2,318	67.1	6 (2–11)
Public	1,139	32.9	1 (1–7)
Child’s gender			
Male	1,843	51.2	5 (1–11)
Female	1,759	48.8	4 (1–10)
Pregnancy intention for the current child			
Wanted at that time	1,896	54.4	4 (1–10)
Wanted later	1,220	35	5 (1–11)
Not at all	368	10.5	5 (1–13)
Ethnic group			
Kikuyu	1,094	31.6	3 (1–8)
Luhya	583	16.8	5 (1–12)
Luo	563	16.2	6 (1–12)
Kamba	712	20.6	4 (1–11)
Other	513	14.8	5 (1–12)

### Regression analysis

The unadjusted TR shows infants with LBW tended to receive the BCG vaccine much later compared to the NBW infants (Table 2). Infants weighing less than 2000 grams and those weighing between 2000–2499 grams received BCG vaccine later, TR = 7.73 [5.52, 10.82] and TR = 1.22 [0.98, 1.51] respectively when compared to normal birth weight infants. Adjusting for the settlement area, ethnicity, mother’s educational level, gender of the child, place of delivery and whether the child was planned, the TR estimates for infants weighing less than 2000 grams and those weighing between 2000–2499 grams became higher, 8.97 [6.01, 13.39] and 1.44 [1.15, 1.82] respectively. The effect of ethnic group of the mother and place of delivery were also statistically significant. Infants from other ethnic groups tend to receive BCG vaccine much later compared to the Kikuyu. Infants delivered at a public health facility received BCG vaccine much earlier (TR = 0.48 [0.44, 0.53]) compared to those delivered at a private health facility. Other variables included in the model did not attain the required level of significance.

**Table 2:** **Unadjusted and adjusted lognormal models estimating the effects of child birth weight on timing of the BCG vaccination**

	**Model 1**			**Model 2**		
	**Unadjusted**			**Adjusted**		
	**Time ratio**	**95% CI**	**Overall P-value**	**Time ratio**	**95% CI**	**Overall P-value**
Child’s birth weight (ref: NBW (weight > =2500 grams))						
2000-2499 grams	1.22	[0.98,1.51]	<0.0001	**1.44**	**[1.15,1.82]**	**<0.0001**
<2000 grams	**7.73**	**[5.52,10.82]**		**8.97**	**[6.01,13.39]**	
Settlement area (ref: Korogocho)						
Viwandani				0.91	[0.82,1.01]	0.079
Ethnic group (ref: Kikuyu)						
Luhya				**1.16**	**[1.02,1.32]**	**<0.0001**
Luo				**1.22**	**[1.07,1.40]**	
Kamba				**1.27**	**[1.11,1.44]**	
Others				**1.31**	**[1.14,1.51]**	
Mother’s level of education (incomplete primary)						
Completed primary				0.91	[0.81,1.02]	0.1773
Secondary+				0.90	[0.80,1.02]	
Pregnancy wanted ness (ref: wanted now)						
Wanted later				1.05	[0.95,1.16]	0.1492
Not at all				1.16	[0.99,1.35]	
Child’s gender (ref: male)						
Female				0.95	[0.87,1.03]	0.221
Type of facility (ref: private HF)						
Public HF				**0.48**	**[0.44,0.53]**	**<0.0001**
N	3602			3185		

Estimates of a significance p < 0.05 are in bold writing.

Model 3 and 4 shows significant interactions of birth weight with gender and place of delivery respectively. From Table 3, the effect of low birth weight (those weighing between 2000–2499 grams) among female was 1.17 [0.89, 1.54] which is 0.6-times that of males 1.95 [1.31, 2.90]. The effect of low birth weight (those weighing <2000 grams) on female was estimated at 8.82 [4.95, 15.71] which was 0.97-times that of boys. Model 3 shows significant birth weight and place of delivery interaction. The effect of low birth weight (those weighing between 2000–2499 grams) among infants delivered in a public health facility was estimated as 1.79 [1.27, 2.54] which is 1.52-times that of infants born in a private health facility (1.18 [0.88, 1.59]). The effect of low birth weight (those weighing <2000 grams) among infants delivered in a public health facility was estimated as 14.41[10.06, 20.64], 3.94-times that of low birth weight (those weighing <2000 grams) infants delivered in a private health facility (3.66[1.78, 7.50]).

**Table 3:** **Interactions lognormal models estimating the effects of child birth weight on timing of the BCG vaccination**

	**Model 3**			**Model 4**		
	**HF interaction**			**Sex interaction**		
	**Time ratio**	**95% CI**	**Overall P-value**	**Time ratio**	**95% CI**	**Overall P-value**
Male effect on LBW(2000–2499)				**1.95**	**[1.31,2.90]**	0.1144
Female effect on LBW(2000–2499)				1.17	[0.89,1.54]	
Male effect on LBW(<2000)				**9.10**	**[5.28,15.68]**	
Female effect on LBW(<2000)				**8.82**	**[4.95,15.71]**	
Effect of private health facility on LBW (2000–2499)	1.18	[0.88,1.59]	0.0009			
Effect of public health facility on LBW (2000–2499)	**1.79**	**[1.27,2.54]**				
Effect of private health facility on LBW (<2000)	**3.66**	**[1.78,7.50]**				
Effect of public health facility on LBW (<2000)	**14.41**	**[10.06,20.64]**				
N	3185			3185		

Estimates of a significance p < 0.05 are in bold writing.

## Discussion

The importance of BCG immunization in sub-Saharan Africa remains well accepted as it is a proven and cost-effective method of conferring immunity against tuberculosis and reducing the risk of outbreak of the disease. The coverage and timeliness of immunizations are the two key indicators of the population level protection against specific diseases. In this study, although coverage with BCG was above 95%, timeliness varied significantly among specific sub-groups. We found that infants born with low birth weight received the BCG vaccine much later than the normal birth weight infants. In particular, the low birth weight (those weighing <2000 grams) infants received the vaccine eight times later than the normal birth weight infants. Although there have been concerns about administering BCG in pre-term and low birth weight babies [19], evidence indicates a normal immune response to vaccine in low birth weight infants [20,21]. The World Health Organization recommends that pre-term infants should be vaccinated at 40 weeks, and this policy may underlie the delay in BCG administration to low birth weight infants even when delivered at full-term [22]. In Kenya, it is common practice in health facilities to delay immunization of the low birth weight infants, but this delay, if in excess, could have negative health consequences especially if the infant is exposed to tuberculosis before getting the BCG vaccine [22]. Studies have shown that BCG also has beneficial non-specific effects [14]. Low birth weight infants would not benefit from these during the early part of their life, if BCG immunization is delayed.

The tendency for late immunization among infants from the non-Kikuyu ethnic groups in Kenya has been reported previously and is consistent with the lower utilization of health services among non-kikuyu ethnic groups as observed in other studies [5,23]. Early receipt of the BCG vaccine by infants born in public health facilities is also consistent with reports from other parts of Sub Saharan Africa [24], which may be due to better vaccine supply in these facilities compared to the private health facilities in the underserved urban areas [25]. In addition, adherence to immunization schedules is required at public health facilities and may account for the early administration of BCG compared.

We assessed the effect of gender of the child in association with birth weight and BCG timing; and found a non- significant interaction, but results showed more delays in males as compared to female. The reasons for the gender difference in the timing of BCG immunization among low birth weight infants are not clear. Further studies are required to determine if this delay is related to parent or caregiver choices associated with low birth weight infants. This is important as studies in West Africa have shown beneficial effects of BCG to be more pronounced in female than male infants [26-28].

The interaction between birth weight and place of delivery where low birth weight infant delivered in public health facilities were immunized much later compared to their counterparts delivered from private health facilities. This is in contrast to the normal birth weight infants delivered from the public health facilities who get immunized earlier. This may be due to the better trained health workers in public health facilities intentionally delaying BCG immunization to low birth weight infants as a precaution, but which staff of private health facilities in the slum may not do.

This study was conducted in two urban slums in the Nairobi and may therefore be indicative of the BCG status of low birth weight infants in similar settlements in Kenya. One limitation of the study is the large number of infants who had to be excluded due to the absence of documented birth weight or immunization status. The exclusion eliminated the effects of recall bias in cases where there is no documentation on birth weight or BCG immunization date. About 32% of the infants and a further 2% were omitted from the analysis due to lack of documentation of birth weight and/or BCG date respectively. The exclusions is supplemented by the large sample size for the analysis due to the longitudinal nature of the study. In addition, the absence of information from caregivers regarding the reasons for delays in BCG immunization would have clarified the extent of service-related delays.

## Conclusions

This study shows that low birth weight infants in Nairobi urban informal settlements receive BCG immunization much later than normal birth weight infants with place of delivery as the main factor of influence. This study contributes to addressing the gap in evidence on timeliness of immunization in urban informal settlements in Kenya as well as other similar settings in low and middle income countries. Additional research should be conducted to further evaluate the timing of the other routine childhood immunizations in similar settings as well as rural and formal urban settlements. These studies should evaluate the effects of immunization delays on overall child health.
